# Glymphatic system: a self-purification circulation in brain

**DOI:** 10.3389/fncel.2025.1528995

**Published:** 2025-02-12

**Authors:** Siying Chen, Huijing Wang, Lini Zhang, Yingying Xi, Yiying Lu, Kailin Yu, Yujie Zhu, Izmailova Regina, Yong Bi, Fang Tong

**Affiliations:** ^1^School of Health Science and Engineering, University of Shanghai for Science and Technology, Shanghai, China; ^2^Institute of Wound Prevention and Treatment, School of Fundamental Medicine, Shanghai University of Medicine & Health Sciences, Shanghai, China; ^3^Department of Neurology, Shanghai University of Medicine & Health Sciences Affiliated Zhoupu Hospital, Shanghai, China

**Keywords:** glymphatic system, cerebral spinal fluid, astrocyte, AQP4, brain disease

## Abstract

The glymphatic system theory introduces a new perspective on fluid flow and homeostasis in the brain. Here, cerebrospinal fluid and interstitial fluid (CSF-ISF) moves from the perivascular spaces (PVS) of arteries to those of veins for drainage. Aquaporin-4 (AQP4) plays a crucial role in driving fluid within the PVS. The impairment to AQP4 is closely linked to the dysfunction of the glymphatic system. The function of the glymphatic system is less active during waking but enhanced during sleep. The efficiency of the glymphatic system decreases with aging. Damage to the glymphatic system will give rise to the development and progression of many brain diseases, such as Alzheimer’s disease (AD), Parkinson’s disease (PD), chronic traumatic encephalopathy (CTE), and vascular dementia (VaD). Here, we reviewed previous research associated with the glymphatic system, including its concepts, principles, and influencing factors. We hypothesize that AQP4 could be a target for the prevention and treatment of certain brain diseases through the regulation on the glymphatic system.

## 1 CSF-ISF circulation and glymphatic system in brain

**FIGURE 1:**
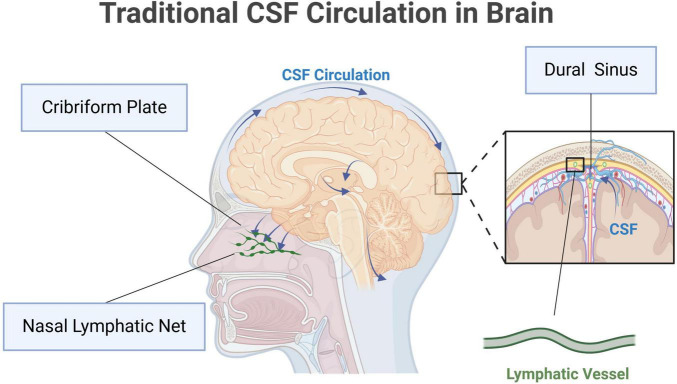
Traditional cerebrospinal fluid (CSF) circulation in brain. Original image by Illustrator (https://app.biorender.com/), modified by the author. CSF is produced by the choroid plexus and flows from the lateral ventricles into the third ventricle through the interventricular foramen. It then moves into the fourth ventricle via the mesencephalic aqueduct. From the fourth ventricle, CSF enters the subarachnoid space through the median and lateral apertures. In the subarachnoid space, CSF circulates around the brain and spinal cord, eventually absorbed into the dural sinuses, primarily the superior sagittal sinus, through the arachnoid granulations. The meningeal lymphatic vessels near the dural sinuses also take part in the absorption of CSF. Additionally, CSF can travel along the olfactory nerve through the lamina cribrosa to the nasal mucosa, where it is absorbed by cervical lymphatic vessels.

Cerebrospinal fluid is a kind of clear, colorless fluid, substantially occupying the space between the pia and arachnoid. CSF is secreted by the choroid plexus within the ventricles, and eventually absorbed through the venous sinuses (Bubeníková et al., 2023; Soytürk et al., 2021; Trillo-Contreras et al., 2019). It nourishes and protects the central nervous system (CNS). CSF circulation starts from the lateral ventricles, and flows through the interventricular foramina into the third ventricle. Then, it travels via the cerebral aqueduct to the fourth ventricle. CSF enters the subarachnoid space through the median foramen and the two lateral foramina, and infiltrates through the arachnoid granulations into the dural sinuses (Wright et al., 2012). The meningeal lymphatic vessels near the dural sinuses also absorb a small amount of CSF. Additionally, CSF can flow through the sieve plate around the olfactory nerve, and is eventually absorbed into the cervical lymphatic vessels in the nasal mucosa (Chae et al., 2024). The traditional CSF circulation is summarized in Figure 1.

An increasing number of studies describe new findings on the understanding of the CSF pathway. CSF can flow into the parenchyma from the PVS of the cortex, where the subarachnoid space widens locally. This widening allows CSF to move efficiently and reach the pericellular space (ECS) in the deep brain (Bedussi et al., 2017). During this process, CSF gradually migrates into the ISF at the PVS of small arteries, continuously exchanging matter with blood vessels. This process is known as CSF-ISF circulation. At the surface layer, the composition of CSF and ISF is similar; however, as CSF flows into deeper brain parenchyma along the PVS, the free interchange between CSF and ISF decreases. The fluid composition gradually changes with substantial interchange between the PVS and brain blood vessels through channels and transporters. In addition to providing the internal environment for neuronal cells, CSF-ISF also maintains the structure of the ECS and stabilizes brain volume (Iliff et al., 2012). If the ECS exceeds the normal range, the structure of neuronal cells may be damaged. Therefore, CSF-ISF flow must be regulated within a specific range.

**FIGURE 2:**
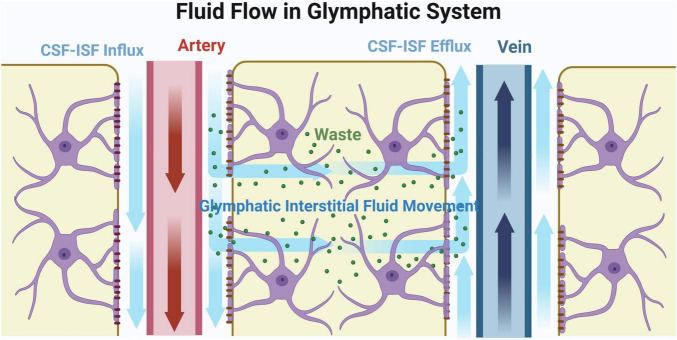
Fluid flow in glymphatic system. Original image by Illustrator (https://app.biorender.com/), modified by the author. Cerebrospinal fluid (CSF) initially flows through the subarachnoid space surrounding the cerebral arteries and into the perivascular spaces (PVS), as indicated by the blue arrow. Upon entering the brain parenchyma, CSF mixes with interstitial fluid (ISF). Aquaporin-4 (AQP4) regulates fluid flow in the PVS, facilitating the transport of metabolic waste products along the PVS to the veins for reabsorption. The interstitial fluid that enters the brain parenchyma eventually exits through the perivenous space, effectively removing metabolic waste products from the brain.

The terminals of the CSF-ISF circulation are small veins which recycle the fluid into blood circulation (Abbott et al., 2018; Menéndez González, 2023a). This pathway was first reported in 2012. A study team injected fluorescent tracers into the cisterna magna of mouse, and found that CSF-ISF circulation acts as a self-purification system for brain (Iliff et al., 2012). Neurotoxic wastes, such as β-Amyloid (Aβ), were observed to be eliminated through CSF-ISF flow (Iliff et al., 2012). This process is similar to the peripheral lymphatic system which recycles excess fluid and clears metabolic wastes produced from tissues. Previously, it was believed that the brain did not contain lymphatic system. However, new findings demonstrate that the function of CSF-ISF circulation at PVS is similar to the lymphatic system. AQP4, an important water-channel protein which is widely expressed on the astrocytic endfeet in brain, plays crucial roles in the regulation of the CSF-ISF circulation. Specifically, AQP4 allows fluid to move smoothly at PVS. Considering the similar function to the lymphatic system and the strong relationship with glia, this newly discovered self-purification system in brain is named glymphatic system. The pathway of fluid flow in the glymphatic system is described in Figure 2.

## 2 AQP4: a central protein of glymphatic system

The AQP4 on the astrocytic endfeet physically wraps the brain blood vessels as an important part of blood-brain barrier (BBB) through binding with α-syntrophin on the perivascular membrane (Hubbard et al., 2015). AQP4 exists in four isoforms: two classical forms, M1 and M23, and two newly-discovered forms, M1x and M23x (Mueller et al., 2023). The ultrastructure of AQP4 is described as orthogonal particle arrays (OAPs) which enhance water permeability and cerebrospinal fluid flow (Hubbard et al., 2015). The organization of OAPs is primarily influenced by the balance between M1 and M23 isoforms. An excessive proportion of M1 can impair the OAPs and in turn reduces the water permeability of AQP4 (Peng et al., 2023). Additionally, translational readthrough generates a C-terminal extended variant of AQP4x which includes two readthrough isoforms, M1x and M23x (Mueller et al., 2023; Sapkota et al., 2019; Sapkota et al., 2022). AQP4x is localized exclusively around BBB, while AQP4-M1 and -M23 are not. Previous studies have demonstrated that AQP4x could enhance AQP4-mediated glymphatic clearance and facilitating Aβ removal in the brain (Sapkota et al., 2019; Sapkota et al., 2022). These findings collectively suggest that it should be AQP4x that maintains the fluid movement in the glymphatic system.

The driving force of CSF-ISF circulation was once thought to be mechanical force from cerebral artery pulsation, which directly pushes fluid to flow at PVS (Garborg, 2024). However, this force alone is not strong enough to maintain the continuous fluid flow in the glymphatic system. Therefore, there must be additional driving forces apart from the artery pulsation. The chemical force from osmosis mediated by AQP4 is considered crucial in the glymphatic system (Asgari et al., 2016; Mestre et al., 2018). Specifically, high blood pressure leads to fluid efflux in the PVS of arteries, and low blood pressure causes influx at veins. This process creates an artery-vein gradient of osmotic pressure, driving fluid flow at PVS. AQP4 provides an efficient channel for fluid movement and is thought to be the origin of the chemical force. The loose fibrous matrix of the PVS provides a low-resistance pathway, allowing fluid to move smoothly. Osmosis is strong enough to drive fluid to permeate efficiently from the PVS to ECS, maintaining ECS structures (Pizzo and Thorne, 2017). The fluid influx and the high permeability of veins facilitate protein reabsorption and elimination of neurotoxic metabolites. Nonetheless, there are studies challenging the glymphatic hypothesis by demonstrating that solute transport in the brain was not significantly affected by AQP4 deletion as well as cardiorespiratory arrest (Smith et al., 2017). They concluded that the perivascular solute transport was facilitated only by the simple diffusion independent of AQP4 (Smith et al., 2017). As far as we are concerned, we support the existence of the glymphatic system and its function of solute transport as well as self-clearance. At least, the general fluid movement in the brain is hugely affected by AQP4. Numerous studies have proved that the impairment on AQP4 may decrease the efficiency of CSF-ISF flow (Hubbard et al., 2015; Mestre et al., 2020). AQP4 deficiency in wildtype animals contributed to a 70% reduction in cerebral fluid flow (Iliff et al., 2012; Peng et al., 2023). In the animals with medial cerebral artery ligation (MCAO), AQP4 knockout reduced CSF influx into brain tissue. Mice without AQP4 demonstrated decreased CSF flow after MCAO, and did not develop cerebral edema within 15 min post-MCAO (Mestre et al., 2020). In a cerebral infarction model, TGN-020, an AQP4 inhibitor, alleviated CSF-derived cerebral edema (Pirici et al., 2017). Furthermore, AQP4 pathologies have been found to be associated with the deposition of neurotoxic proteins such as Aβ and α-synuclein (α-syn), suggesting that the development and progression of neurodegenerative diseases should be triggered by the disruption of glymphatic system. All the evidence above collectively implies the existence of glymphatic system. We believe that its underlying mechanisms will be further clarified as new findings emerge in the future.

**FIGURE 3:**
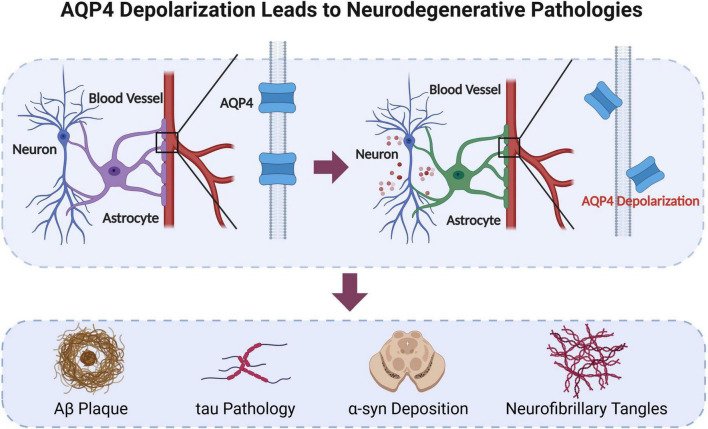
Aquaporin-4 (AQP4) depolarization leads to neurodegenerative pathologies. Original image by Illustrator (https://app.biorender.com/), modified by the author. AQP4 is a crucial protein for maintaining the glymphatic system in the brain. When AQP4 becomes depolarized, the clearance function of glymphatic system decreases. This leads to a series of pathological changes, such as Aβ plaque, α-synuclein deposition, tau pathology and neurofibrillary tangles.

The polarized distribution of AQP4 surround BBB is mediated by the bonds between AQP4x and α-syntrophin. Transgenic mice without α-syntrophin demonstrated AQP4 depolarization around BBB (Simon et al., 2022). While mice without AQP4x reduced α-syntrophin expression by 40%, and the perivascular localization of α-syntrophin (Mueller et al., 2023; Palazzo et al., 2019). AQP4 polarization is found to be crucial for maintaining the function of the glymphatic system. The clearance rate of glymphatic system significantly decreases with the loss of polarized distribution (Li et al., 2022; Liang et al., 2015; Lu et al., 2021). AQP4 depolarization will lead to a series of neuropathological changes which is illustrated in Figure 3. AQP4 impairment along with neurodegenerative pathologies occur in many brain diseases, such as cerebral infarction, Alzheimer’s disease, and traumatic brain injury (TBI) (Mader and Brimberg, 2019).

## 3 Assessment of glymphatic system

The assessment of the glymphatic system commonly involves using tracers along with *in vivo* dynamic imaging. In the first research on the glymphatic system, mice were injected with a fluorescent tracer into the cisterna magna (Iliff et al., 2012). This allowed for convenient observation of fluid diffusion on the brain surface under two-photon microscopy. After the brain was sectioned into slices, the fluorescent tracer was observed in deep brain tissue along the PVS of arteries and was absorbed into blood circulation through veins (Simon et al., 2022). By injecting a paramagnetic contrast agent, the CSF-ISF flow in the PVS and the CSF-ISF exchange in brain parenchyma can be effectively observed using magnetic resonance imaging (MRI) (Simon et al., 2022). The gold standard technique for identifying glymphatic circulation involves intrathecal injection of a gadolinium contrast agent with contrast-enhanced magnetic resonance imaging (DCE-MRI) (Palazzo et al., 2019). Compared with two-photon microscopy, MRI has the advantage of recording fluid diffusion throughout the entire brain and is non-invasive (Lu et al., 2021). However, the resolution of MRI is not high enough to detect fluid movement at the microstructural level of the brain.

Diffusion tensor imaging along the perivascular space (DTI-ALPS), an MRI assay, is now used to detect the glymphatic system non-invasively. Unlike projection fibers and association fibers at the lateral ventricular body, DTI-ALPS assesses water diffusivity along the direction of PVS (Taoka et al., 2017). This method does not require a contrast agent, and allows the data collection within a few minutes (Roy et al., 2022). When glymphatic system is damaged, water diffusivity along the PVS will be reduced (Shen et al., 2022). The ALPS index can evaluate the function of the glymphatic system (Zhang et al., 2024). It can reflect the diffusion coefficient along the vascular direction, calculated as the ratio of diffusivity along the PVS to the diffusivity perpendicular to the main fiber bundle and PVS (Chen et al., 2021; Taoka et al., 2022). DTI-ALPS is now used to examine the glymphatic system in the research of age-related cognitive decline. The DTI-ALPS index demonstrates a strong positive correlation with mini-mental state examination (MMSE) and Montreal Cognitive Assessment (MoCA) scores, indicating a link between cognitive decline and reduced water diffusion along the PVS (Liang et al., 2023).

Recently, dynamic infrared imaging has been used to detect the glymphatic system (Keil et al., 2022). This technique is quicker than MRI and two-photon microscopy for capturing images and is also more economical in terms of equipment. However, similar to two-photon microscopy, dynamic infrared imaging cannot observe deep brain parenchyma and requires a craniotomy during the experiment (Keil et al., 2022).

## 4 Glymphatic system during sleep

The function of the glymphatic system is less active during waking but enhanced during sleep (Bojarskaite et al., 2023). This enhancement is due to increased ECS from reduced cell volume during sleep, allowing larger space for CSF-ISF flow (Christensen et al., 2021). AQP4 activity is affected by circadian rhythms, and AQP4 knockdown can eliminate these rhythms in the glymphatic system (Hablitz et al., 2020). Glymphatic flow peaks during the transition to sleep, as shown by tracer injections in experimental animals (Hablitz et al., 2020). There is a strong correlation between the function of the glymphatic system and sleep phases. CSF-ISF flow and glymphatic clearance significantly increase during slow-wave sleep and positively correlate with δ-wave power (Hablitz et al., 2020). Slow vascular movement during Non-Rapid Eye Movement Sleep (NREM) sleep drives fluid flow and solute transport in the PVS. The PVS enlarges after Rapid Eye Movement Sleep (REM) sleep phases, even more during post-sleep awakenings than pre-sleep awakenings, indicating specific PVS dynamics in each sleep cycle phase (Bojarskaite et al., 2023). One night of sleep deprivation significantly increases Aβ deposition in the right hippocampus and thalamus, while high-quality sleep promotes Aβ clearance from the brain (Shokri-Kojori et al., 2018; Wang et al., 2022). Sleep quality is closely linked to AQP4 function and the clearance rate of glymphatic system (Hauglund et al., 2020; Wang et al., 2022). Sleep-deprived mice show AQP4 depolarization (Wang et al., 2022), and AQP4 expression in the amygdala and dentate gyrus (DG) is significantly reduced in mice with fragmented sleep (Vasciaveo et al., 2023). The mechanism by which toxins are removed from the brain remains a topic of ongoing debate. Contrary to earlier studies, recent research has revealed that sleep may not enhance the clearance of brain toxins. Instead, it leads to a significant decrease in the clearance rate by approximately 30% (Miao et al., 2024). This discrepancy may be attributed to variations in experimental procedures used in past studies, necessitating further research for in-depth exploration (Miao et al., 2024).

## 5 Effects of aging on glymphatic system

The function of the glymphatic system decreases with aging. DTI-ALPS has been used to assess the glymphatic system in older adults with normal cognition and found that the DTI-ALPS index decreases with age (Benveniste et al., 2019; Park et al., 2023). During aging, the expression of glial fibrillary acidic protein (GFAP) is significantly enhanced in astrocytes, along with AQP4 depolarization at the PVS, leading to a decrease in CSF influx (Hablitz and Nedergaard, 2021). Decreased glymphatic function due to aging may be a causative factor in age-related neurodegeneration. The clearance of Aβ is reduced by 40% in aged mice compared to young mice (Mestre et al., 2022).

The decreased function of glymphatic system is ascribed to vascular remodeling during aging. Aging impairs the elasticity of brain blood vessels and diminishes the amplitude of arterial pulsations, giving rise to cerebral vascular sclerosis. Aging progressively inactivates the Notch3 signaling pathway in the cerebral vasculature, disrupting the regulation of calcium signaling and the contractile function of these vessels (Romay et al., 2024), which may cause vasodilatation, tortuosity, and microaneurysms in brain. Vascular remodeling eventually reduces CSF-ISF flow, inhibiting the function of glymphatic system to clear the wastes from the brain (Iliff et al., 2014; Romay et al., 2024; Zhou et al., 2020). As a result, the accumulation of neurotoxic wastes in the brain parenchyma will eventually lead to neurodegenerative pathologies.

Aging-induced immune dysregulation can impair the glymphatic system, in addition to causing vascular remodeling. CC-chemokine receptor 7 (CCR7), a key organizer of the primary immune response, is expressed by B lymphocytes, mature dendritic cells (DCs), and several T-cell sub-populations (Förster et al., 1999; Förster et al., 2008). Aging-induced hematopoietic CCR7 deficiency can damage glymphatic system and harm spatial memory (Da Mesquita et al., 2021). Reduced CCR7 expression in T cells with aging disrupts meningeal immunity and affects the function of astrocytes and microglia in the aging brain. As a result, the expression of polarized AQP4 in astrocytes decreases (Da Mesquita et al., 2021; Formolo et al., 2023). CCR7 deficiency also causes significant changes in gene expression in the blood endothelial cells (BEC) of brain. Together with reduced AQP4 expression, these changes can impair the function of glymphatic system and causes neurodegenerative pathologies (Da Mesquita et al., 2021).

## 6. Glymphatic system and brain diseases

### 6.1 Alzheimer’s disease

The onset and progression of AD are closely linked to the impairment of the glymphatic system. This theory not only deepens our understanding of AD pathogenesis but also highlights new therapeutic targets for its treatment (Silva et al., 2021). The representative neuropathological changes of AD include the formation of p-tau, Aβ plaques, and neurofibrillary tangles which leads to irreversible cognitive damage (Simon et al., 2022). The key issue in AD development is the imbalance between Aβ production and clearance (Zeppenfeld et al., 2017). AD patients have a slower rate of Aβ clearance, while Aβ production remains similar (Li et al., 2024). The disruption of glymphatic system is linked with an abnormal size of PVS, and in turn, impairs Aβ clearance, leading to the development and progression of AD (Ang et al., 2024; Park et al., 2024). The research team of Iliff et al. (2012) injected Aβ1-40 through the cisterna magna and observed Aβ clearance in real time. They found that Aβ clearance was reduced by 55% in AQP4 knockout mice. In APP/PS1 mice, AQP4 knockout does not affect Aβ production but leads to a 25–50% increase in soluble and insoluble Aβ accumulation in the brain parenchyma (Zeppenfeld et al., 2017). These results are due to reduced CSF-ISF flow caused by AQP4 absence, which reduces Aβ clearance through the BBB. Some autopsy studies of AD patients have found a depolarized distribution of AQP4 around blood vessels (Zeppenfeld et al., 2017). Studies based on immunohistochemistry have demonstrated that AQP4 is abundantly expressed in astrocyte endfeet around Aβ plaque areas, particularly around vessels with amyloidosis (Hoshi et al., 2012; Moftakhar et al., 2010). Perivascular AQP4 depolarization has a greater impact on Aβ deposition than the overall deletion of the AQP4 gene in AD progression (Pedersen et al., 2023). Furthermore, AQP4 depolarization commonly occurs alongside astrogliosis, implying a potential link between the two pathological changes (Li et al., 2024). Since CSF-ISF can move through the “olfactory nerve-nasal mucosa-nasal lymph” pathway, Aβ may deposit in the olfactory nerve and cause axonal injury. Interestingly, some AD patients demonstrate smell damage in addition to cognitive decline and motor dysfunction (Chae et al., 2024).

### 6.2 Parkinson’s disease

Parkinson’s disease is a neurodegenerative disorder characterized by the abnormal accumulation of α-synuclein (α-syn) in neurons and the loss of dopaminergic neurons in the substantia nigra compacta (Hoshi et al., 2012). Under normal physiological conditions, there is a dynamic balance between the production and degradation of α-syn. The glymphatic system may play a crucial role in clearing extracellular α-syn and its isomers from the brain (Moftakhar et al., 2010). However, the relationship between the glymphatic system and the progression of PD remains unclear (Pedersen et al., 2023).

Limited evidence suggests that the damage to glymphatic system exacerbates α-syn deposition and decreases dopaminergic neurons (Menéndez González, 2023b; Yao et al., 2024). AQP4 is also implicated in the progression of PD. Autopsy results have shown that AQP4 expression levels are negatively correlated with α-syn content in the neocortex of PD patients (Wang et al., 2024). In mice with the overexpression of human A53T-α-syn, AQP4 deficiency accelerated the pathological deposition of α-syn and promoted dopamine neuron loss (Cui et al., 2021). DTI-ALPS provides neuroimaging evidence of glymphatic system dysfunction in PD patients (Gu et al., 2023; Si et al., 2022). The DTI-ALPS index is negatively correlated with cognitive decline in PD patients (Si et al., 2022). The decrease in the DTI-ALPS index typically begins in the left hemisphere of the brain and spreads to the right hemisphere as PD progresses (Si et al., 2022).

Sleep disorder is a common clinical manifestation in PD patients. As we have mentioned in Part 4, the glymphatic system could be enhanced during sleep (Nedergaard and Goldman, 2020). Thus, it is hypothesized that impaired glymphatic function caused by sleep disorders is a leading cause of PD (Massey et al., 2022). Specifically, sleep disorders disrupt CSF-ISF rhythmicity and decrease α-syn clearance in the glymphatic system, harming dopaminergic neurons (Hablitz et al., 2020; Stefani and Högl, 2020).

### 6.3 Chronic traumatic encephalopathy

Traumatic brain injury is a common occurrence in sports, accidental falls, and road traffic accidents. Many TBI survivors develop clinical manifestations similar to AD and PD years after the injury, including mood changes, motor impairments, and cognitive deficits (Wilson et al., 2017). These chronic neurodegenerative conditions associated with TBI are collectively referred as Chronic traumatic encephalopathy (CTE). Over 80% of brain donors from the two largest brain banks for CTE were professional athletes in American football, Canadian football, soccer, rugby, hockey, and boxing (Castellani, 2020). This suggests that CTE is prevalent in professional contact sports. Additionally, CTE has been observed in amateur athletes of contact sports (Adams et al., 2018). While athletes often suffer from concussions during their careers, CTE involves more than repeated concussions. The risk of CTE is closely related to the frequency and intensity of head trauma, with 30% of individuals experiencing repetitive head impacts (RHI) eventually being diagnosed with CTE (Asken et al., 2017; McKee et al., 2015). The findings of CTE neuropathology date back to an autopsy of a boxer in 1954, who exhibited dementia and dyskinesia after retirement (Brandenburg and Hallervorden, 1954). The autopsy revealed cortical atrophy, flattening of the corpus callosum, ventricular dilatation, perforation of the septum pellucidum, and disappearance of the substantia nigra in the midbrain. Microscopically, neurofibrillary tangles (NFTs) were observed in the brain tissue, along with a decrease in the number of neurons (Brandenburg and Hallervorden, 1954). With the accumulation of autopsy cases, these lesions are now considered common in long-term survivors of TBI. Additionally, tau pathology is observed in the brains of CTE patients (McKee et al., 2015). Immunohistochemical staining has shown p-tau deposits in NFTs, neurons, and astrocytes, distributed along the periphery of small vessels in the deeper parts of the brain (Johnson et al., 2012). Furthermore, 95% of CTE cases exhibit varying degrees of Aβ plaques in the brain (McKee et al., 2023), and some studies have also found dilated PVS in the white matter (McKee et al., 2015). The pathogenesis of CTE may be influenced by lesions in the glymphatic system. A single TBI can cause a loss of polarized distribution of perivascular AQP4, which subsequently impairs the clearance function of the glymphatic system (Iliff et al., 2014). In a model of repetitive moderate TBI, ipsilateral hemispheric CSF flow and clearance rate were impaired the day after the injury, and the glymphatic system lost 60% of its function until 28 days post-injury (Kress et al., 2014). This impairment could lead to increased aggregation of tau proteins in the brain and subsequent neurodegeneration (Jessen et al., 2015). AQP4 knockout can increase neuroinflammation post-TBI and affect post-injury cognitive recovery (Chiu et al., 2016). The key pathophysiological processes of CTE induced by repeated mild TBI (rmTBI) may include: (1) Mechanical external force damages the blood-brain barrier and causes the loss of AQP4 polarization around blood vessels, significantly decreasing the efficiency of CSF-ISF circulation; (2) Astrocyte reaction in the brain parenchyma further increases the resistance of CSF-ISF flow; (3) A large amount of neurotoxic metabolites accumulate in the brain, leading to chronic neuroinflammation, neurodegeneration, emotional changes, cognitive and memory impairment, and the onset of CTE (Berry et al., 2020; Sullan et al., 2018).

### 6.4 Vascular dementia

The impairment on glymphatic system caused by cerebral infarction has been observed in rodent and non-human primate models of stroke (Goulay et al., 2017), which is considered to be closely associated with the progress of Vascular dementia (VaD). AQP4 demonstrated depolarization at 150 min post-MCAO, and experimental animals show reduced perivascular AQP4 expression (Venkat et al., 2017; Zhang et al., 2022). The impairment will finally cause the accumulation of neurotoxic wastes in both affected and surrounding brain regions, and proceed to neurodegeneration and cognitive impairment (Ji et al., 2021). Besides, cerebral infarction can also affect the clearance of neuroinflammatory mediators. For instance, IL-6, produced in the core of cerebral infarction, is typically discharged through the glymphatic system. The reduced glymphatic function hinders the removal of IL-6, potentially exacerbating the neuroinflammatory response (Yang et al., 2024). Ipsilateral cortical CSF flow decreases at 3 h post-MCAO but is restored at 24 h post-ischemia-reperfusion (Lin et al., 2020; Lyu et al., 2021). A total of 7 weeks after MCAO, it was observed that the fluorescent tracer in the infarct core leaked through the glial scar and traveled along the perivascular space (Zbesko et al., 2018). In summary, dysfunction of the glymphatic system induced by infarction leads to protein aggregation and misfolding, and then gives rise to local inflammation, neuronal loss, and ultimately, dementia (Nedergaard and Goldman, 2020).

## 7 Conclusion

**FIGURE 4:**
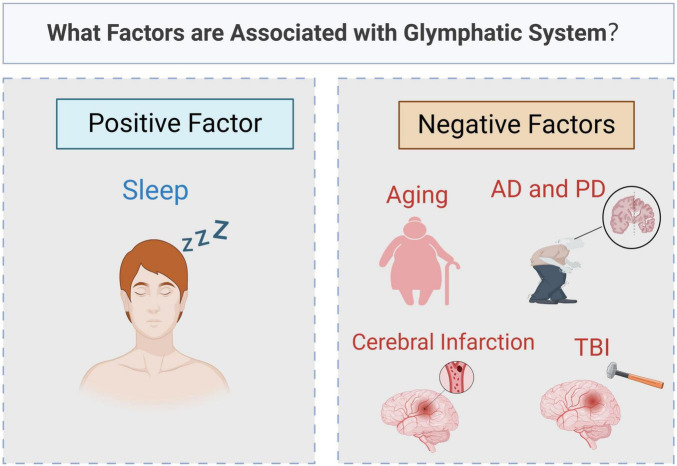
What factors are associated with glymphatic system? Original image by Illustrator (https://app.biorender.com/), modified by the author. The function of glymphatic system is affected by many factors. Sleep is a positive factor, boosting activity and helping to clear waste from the brain. In contrast, aging and brain diseases, such as Alzheimer’s disease (AD), Parkinson’s disease (PD), traumatic brain injury (TBI) and cerebral infarction, can lead to a decline in its function.

The proposed theory of the glymphatic system provides new insights into CSF-ISF circulation and brain homeostasis. In the glymphatic system, CSF-ISF flows from the PVS of arteries to those of veins for drainage. The glymphatic system is essential for maintaining CNS function. AQP4 is the primary protein driving CSF-ISF circulation within the PVS of the glymphatic system. In addition to the mechanical force from cerebrovascular pulsation, osmotic forces generated by AQP4 also facilitate fluid flow in the glymphatic system. This system plays a important role in waste removal in the CNS, with its function influenced by multiple factors (Figure 4). Aging negatively affects glymphatic function, while regular sleep enhances glymphatic activity and supports long-term brain health. Redistribution or decreased expression of AQP4 impairs glymphatic function and is closely associated with neurodegenerative diseases such as AD, PD, and CTE. We hypothesize that AQP4 could therefore be a target for preventing and treating certain brain diseases.
